# GLUT1 exacerbates trophoblast ferroptosis by modulating AMPK/ACC mediated lipid metabolism and promotes gestational diabetes mellitus associated fetal growth restriction

**DOI:** 10.1186/s10020-024-01028-x

**Published:** 2024-12-20

**Authors:** Qin Zhang, Xi Yuan, Xiaojin Luan, Ting Lei, Yiran Li, Wei Chu, Qi Yao, Philip N. Baker, Hongbo Qi, Hui Li

**Affiliations:** 1 Department of Obstetrics, The First Affiliated Hospital of Chongqing Medical University, Chongqing, 400016 China; 2 Chongqing Key Laboratory of Maternal and Fetal Medicine, Chongqing Medical University, Chongqing, 400016 China; 3 Department of Obstetrics and Gynecology, Women and Children’s Hospital of Chongqing Medical University, Chongqing, 401147 China; 4 College of Life Sciences, University of Leicester, Leicester, LE1 7RH UK; 5 Department of Hematology, Southwest Hospital, Third Military Medical University, Chongqing, 400038 China

**Keywords:** GLUT1, Ferroptosis, Gestational diabetes mellitus, Fetal growth restriction, AMPK/ACC pathway

## Abstract

**Background:**

Gestational diabetes mellitus (GDM) has been associated with several fetal complications, such as macrosomia and fetal growth restriction (FGR). Infants from GDM associated FGR are at increased risk for adult-onset obesity and associated metabolic disorders. However, the underlying mechanisms of GDM associated FGR remain to be explored.

**Methods:**

We analyzed placentas from GDM patients with FGR for ferroptosis markers and GLUT1 expression. High glucose conditions were established by adding different concentrations of D-Glucose to the 1640 cell culture medium. RSL3 were used to test ferroptosis sensitivity in trophoblast cells. GLUT1 was inhibited using siRNA or its inhibitor WZB117 to assess its impact on ferroptosis inhibition in HTR8/SVneo cell line. Mechanistic studies explored the effects of GLUT1 on AMPK and ACC phosphorylation, which in turn impacted lipid metabolism and ferroptosis. In mouse models, streptozotocin (STZ)-induced GDM was treated with WZB117 and the ferroptosis inhibitor liproxstatin-1 (Lip-1). Finally, AMPK and ACC phosphorylation levels were evaluated in GDM patient samples.

**Results:**

In this study, placentas from GDM patients with FGR showed signs of ferroptosis and upregulation of GLUT1. In cell models, high glucose conditions sensitized trophoblast cells to ferroptosis and induced GLUT1 expression. Interestingly, GLUT1 inhibition significantly suppressed ferroptosis in trophoblast cells under high glucose conditions. Mechanistically, elevated GLUT1 inhibited AMPK phosphorylation and reduced ACC phosphorylation, thereby promoting lipid synthesis and facilitating ferroptosis. In pregnant mice, STZ-induced hyperglycemia led to FGR, and treatment with either the GLUT1 inhibitor WZB117 or the ferroptosis inhibitor Lip-1 alleviated the FGR phenotype. Moreover, in vivo elevation of GLUT1 increased ferroptosis markers, decreased AMPK/ACC phosphorylation, and resulted in altered lipid metabolism, which likely contributed to the observed phenotype. Finally, placental samples from GDM patients showed reduced AMPK and ACC phosphorylation.

**Conclusions:**

Our findings suggest a potential role of ferroptosis in GDM associated FGR and indicate that the dysregulated GLUT1-AMPK-ACC axis may be involved in the pathogenesis of GDM associated FGR in clinicals.

**Supplementary Information:**

The online version contains supplementary material available at 10.1186/s10020-024-01028-x.

## Background

Gestational diabetes mellitus (GDM) complicates approximately 7–14% of pregnancies worldwide (McIntyre et al. 2019). Fetal growth restriction (FGR) is less commonly associated with GDM compared to macrosomia, but it still represents a significant concern. The incidence of GDM-associated FGR is estimated to range from 5 to 10% of GDM pregnancies, though this can vary depending on the severity of GDM and the adequacy of metabolic control (Lowe et al. 2019). FGR in the context of GDM is associated with serious perinatal and long-term consequences. Infants born with FGR due to GDM are at increased risk for preterm birth, perinatal asphyxia, hypoglycemia, and other neonatal complications (Melamed et al. 2021). Long-term consequences include a higher likelihood of developing metabolic syndrome, type 2 diabetes, and cardiovascular disease later in life (Melamed et al. 2021; Fung and Zinkhan 2021). Additionally, these infants often experience neurodevelopmental impairments, including cognitive delays and an increased risk of conditions such as cerebral palsy (Check et al. 2024). The dual burden of GDM and FGR represents a significant public health challenge (Jiang et al. 2022), contributing to increased socioeconomic burden due to the need for intensive monitoring, potential early delivery, and neonatal intensive care for affected infants. The long-term health consequences for children born with FGR also lead to ongoing healthcare needs (Sacchi et al. 2020), highlighting the broader impact on healthcare resources. Thence, understanding the mechanisms underlying this condition is crucial for developing effective interventions to improve maternal and fetal outcomes.

Placental insufficiency is a critical aspect of GDM pathophysiology that contributes to the increased risk of fetal and maternal complications. In GDM, placental insufficiency can occur due to vascular dysfunction, characterized by impaired remodeling of the spiral arteries and increased resistance in the uteroplacental circulation. This leads to reduced perfusion of the placenta, limiting the delivery of oxygen and nutrients to the fetus, which can result in chronic hypoxia and nutrient deprivation. Such complications are more pronounced in cases where GDM is poorly controlled and are central to the development of FGR (Lappas et al. 2011; Gauster et al. 2012). This stress is primarily induced by hyperglycemia that overwhelms the placental antioxidant defenses (Li et al. 2020a; Joo et al. 2021). The excessive ROS levels contribute to the activation of inflammatory pathways, further impairing placental function and increasing the risk of adverse pregnancy outcomes (Schliefsteiner et al. 2017).

While oxidative stress is acknowledged as a key driver in GDM pathology, the specific mechanisms by which it leads to GDM-associated FGR remain underexplored and require further investigation. Recent studies highlight the significant contributions of oxidative stress and ferroptosis in the pathophysiology of GDM. Obesogenic diets during pregnancy were shown to disrupt placental iron handling and promote oxidative damage, exacerbating ferroptotic signaling and impairing fetal growth (Zaugg et al. 2024). Moreover, mitochondrial dysfunction, such as SIRT3 deficiency, exacerbates ferroptosis by reducing GPX4 activity, linking impaired mitochondrial regulation to heightened ferroptosis susceptibility under hyperglycemic conditions (Han et al. 2020). Lastly, placental tissues in GDM pregnancies exhibit increased sensitivity to ferroptosis inducers due to diminished antioxidant defenses, explaining the severity of placental injuries in such cases (He et al. 2022).These studies collectively highlight the importance of ferroptosis in FGR; however, further research is needed to fully elucidate the role of ferroptosis in FGR within the context of GDM pregnancies.

Glucose transporter 1 (GLUT1) plays a crucial role in glucose uptake and transfer in placental tissues, ensuring adequate fetal growth and development (Borges et al. 2019). Previous studies have shown that GLUT1 expression is upregulated in term pregnancies complicated by type 1 pregestational diabetes mellitus, potentially affecting pregnancy outcomes (Balachandiran et al. 2021; Stanirowski et al. 2022). Although this initial upregulation of GLUT1 may act as a compensatory mechanism to manage altered glucose levels, excessive upregulation can overwhelm the cell’s antioxidant defenses, intensifying oxidative stress by generating more oxidative byproducts of glucose metabolism (Cheng et al. 2022). This heightened GLUT1 activity may further aggravate ferroptosis by increasing oxidative stress, impairing placental function, nutrient transfer, and ultimately contributing to FGR. Studies have shown that increased GLUT1 expression in GDM placentas may be linked to altered metabolic conditions, such as reduced activity of AMP-activated protein kinase (AMPK), which is critical for maintaining cellular energy homeostasis and regulating lipid metabolism (Yamauchi et al. 2002; Steinberg and Hardie 2023). AMPK activation normally inhibits acetyl-CoA carboxylase (ACC), a key enzyme in fatty acid synthesis, thereby reducing lipid accumulation and protecting against ferroptosis (Pang et al. 2021). However, the precise connection between GLUT1 dysregulation and the AMPK/ACC pathway in contributing to trophoblast ferroptosis and GDM-associated FGR remains unclear and warrants further investigation.

In this study, we aimed to investigate the role of GLUT1-mediated ferroptosis in fetal growth restriction (FGR) within a GDM condition, both in vivo and in vitro and to delve deeper into the underlying molecular mechanisms. We hypothesized that GLUT1 upregulation may lead to dysregulation of the AMPK/ACC pathway and subsequent trophoblast cell ferroptosis, contributing to FGR. Our findings are expected to provide new insights into ferroptosis involved in GDM-associated FGR, offering potential targets for early screening and intervention.

## Methods

### Patient recruitment and sample collection

A total of GDM-complicated (n = 15) and normal pregnancies (n = 15) were recruited from the Department of Obstetrics and Gynecology in the First Affiliated Hospital of Chongqing Medical University. GDM was diagnosed from a 75-g oral glucose tolerance test (OGTT) conducted between gestational weeks 24 and 28 in accordance with the International Association of the Diabetes and Pregnancy Study Groups (IADPSG) criteria (International Association of D et al. 2010). The exclusion criteria included pre-existing diabetes or a family history of diabetes. The study was approved by the Ethics Committee of the First Affiliated Hospital of Chongqing Medical University (ID: 2024-291-01) and informed consent was obtained from all subjects. All procedures were performed in accordance with the principles stated in the Declaration of Helsinki. Their detailed clinical characteristics are shown in Table S1.

### Animals

Animal studies were conducted with approval from the Animal Experimental Ethical Committee of Chongqing Medical University (ID: IACUC-CQMU-2024-02026). C57BL/6 J mice (8 weeks old) were purchased from Beijing Vital River Laboratory Animal Technology Co., Ltd. Mice were housed in a stable facility with 12 h light/12 h dark cycles at 25 °C. All mice were stabilized for a week to acclimatize to the experimental environment. All animal procedures conformed to guidelines for the Care and Use of Animals published by the Institutional Animal Ethical Committee. The gestational day (E) 0.5 was defined by the presence of a vaginal plug day. A GDM mouse model was established by streptozotocin (STZ; 100 mg/kg, dissolved in 0.1 mmol/L citrate buffer, pH 4.2–4.5) (Sigma-Aldrich, USA, V900890) intraperitoneally at E12.5 after fasting 12 h. The control mice (n = 7) were injected with the same amount of citrate buffer. The GDM mice were then randomly assigned into three experimental groups, with the assignment process conducted in a blinded manner to minimize bias during treatment allocation. The first group receives only STZ (n = 5). The second group, STZ + Lip1 (n = 5), was treated with the ferroptosis inhibitor Lip1 (10 mg/kg, dissolved in saline) (TargetMol, T2376), and administered intraperitoneally every other day. The third group, STZ + WZB117 (n = 5), received the GLUT1 inhibitor WZB117 (10 mg/kg, dissolved in saline) (MedChemExpress, NJ, USA, HY-19331), also administered intraperitoneally every other day. On gestational day 18, mice were anesthetized with an intraperitoneal injection of 3% pentobarbital sodium solution. Blood was collected from the hearts and centrifuged at 3000 r/min for 15 min. The placenta tissues and vital organs such as the liver, subcutaneous fat, and pancreas were collected after sacrificing animals.

### Glucose tolerance tests

For the glucose tolerance test (GTT), mice were fasted 16 h and then intraperitoneally injected with 2 g/kg body weight of glucose dissolved in saline on the 18th day of pregnancy. Blood glucose levels were then measured from tail vein blood with a glucometer and test strips at 0, 15, 30, 60, and 120 min after injection. After excluding pregnant mice without complete data, a total of 7 mice from the control group were analyzed, 5 mice from the STZ-treated group, 4 mice from the STZ + Lip1-treated group, and 4 mice in the STZ + WZB117-treated group. The results of GTTs were displayed as blood glucose curves and the area under the curve (AUC).

### Cell culture

The immortalized human chorionic trophoblast line HTR8/SVneo cell were obtained from the American Type Culture Collection (ATCC, USA) and cultured in RPMI-1640 (Gibco, USA) supplemented with 10% FBS (PAN, Germany) and 1% penicillin–streptomycin (Gibco, USA) in a humidified incubator with 5% CO2 in air atmosphere at 37 °C. HTR8/SVneo cell line were cultured with D-Glucose (Sigma-Aldrich, USA, G7021) for 24 h to establish a high glucose condition in vitro model. To establish a high-glucose environment at various concentrations in the HTR8/SVneo cell culture, a 20 × stock solution was prepared and diluted by adding 5 μl of the stock solution to 95 μl of complete medium, creating a 1X working solution at the desired concentration. The control group was treated with an equivalent volume of PBS to ensure consistency.

### Transfection

Small interfering (si)-GLUT1 and a negative control siRNA (siNC) were synthesized by GenePharma (Shanghai, China). Four siRNA sequences targeting GLUT1 were used, numbered 637, 780, 1255, and 1471. HTR8/SVneo cells at 60–70% confluency were transfected with 100 nM siRNA in the presence of Lipofectamine 2000 (Thermo Fisher Scientific, USA) in six-well plates following the manufacturer’s protocols. The efficacy of siRNA interference was verified by Western blots, and the two siRNAs showing the strongest interference effects were selected for subsequent experiments.

### Immunohistochemistry

Four-micrometer paraffin sections of placental tissues were cut. Deparaffinization and rehydration of tissue slices were performed using a graduated alcohol series. Antigen repair was achieved by microwave treatment of the sections in 10 mM sodium citrate (pH 6.0) for 15 min. The slices were then incubated with 3% H2O2 for 15 min to inhibit endogenous peroxidase activity. Then, the sections were incubated with primary antibodies cytokeratin 7 (CK7) (1:100; Proteintech, China, 66483-1-Ig), human leukocyte antigen G (HLA-G) (1:100; Proteintech, China, 66447-1-Ig), GLUT1(1:100; Proteintech, China, 21829-1-AP) overnight at 4 °C. Afterward, RT peroxidase was treated with horseradish coupled secondary antibody for 30 min. Finally, the immune complexes were visualized using a diaminobenzidine (DAB) solution. These images were taken on a NanoZoomer S360 Digital slide scanner (HAMAMATSU, Japan, C13220-01). ImageJ software (NIH, Maryland, USA) was used to measure the intensity of positive staining in each sample. Briefly, each field was randomly selected and did not overlap. The Integrated Optical Density (IOD) is divided by the area of the target protein distribution region to calculate the Average Optical Density (AOD). Statistical analysis of AOD values was then performed to compare target protein expression levels across different groups, allowing for quantification of expression differences.

### Immunofluorescence

After deparaffinization and rehydration, antigen retrieval was performed using sodium citrate. After antigen retrieval, tissue sections were incubated with 5% bovine serum albumin (Sigma-Aldrich, USA, A1993) for 60 min at room temperature. The tissues were then incubated with primary antibodies in 5% BSA overnight at 4 °C. The following primary antibodies were used at a dilution of 1:100: GLUT1 (Proteintech), cytokeratin 7 (Proteintech), and 4-HNE (Invitrogen, United States, MA5-27570). Fluorescein isothiocyanate-conjugated goat anti-rabbit and anti-mouse antibodies (1:100; Proteintech, China) were used as secondary antibodies. The nuclei were counterstained with Hoechst (1:100; Solarbio, China, C0031). Images were captured using an Olympus-fluorescence microscope (Olympus Corporation, Japan, Hyper E301). The immunofluorescence data were quantified using ImageJ software, based on five randomly selected fields per slide. The intensity and cell count in each field were measured, and the average intensity per cell was calculated for analysis.

### Perls’ prussian blue iron staining

Iron deposition in placental tissues was analyzed using the Perls’ Prussian Blue Iron Staining (Solarbio, China, G1424), conducted following the experimental protocol outlined in the manufacturer’s instructions. Briefly, after deparaffinization, sections were incubated in Perls' staining solution for 120 min, followed by three washes in distilled water. The sections were then immersed in eosin solution for 15 s and rinsed in distilled water. Dehydration was performed twice with anhydrous ethanol, followed by clearing in xylene and mounting with neutral resin. Stained cells were visualized and captured using an Olympus-fluorescence microscope.

### Hematoxylin and Eosin (H&E) staining

Placentas were fixed with 4% paraformaldehyde and embedded in paraffin, then sectioned into 3-µm sections. Sections were deparaffinized, rehydrated, and stained with hematoxylin for 4 min, followed by staining with Eosin for 20 s, dehydrated twice with absolute ethanol, transparent with an environmentally friendly clearing agent, and mounted. Imaging utilized a NanoZoomer S360 Digital slide scanner (HAMAMATSU). Morphological analysis was performed to observe the placental labyrinth and spongiotrophoblast. The areas of the labyrinth, spongiotrophoblast and total placenta were calculated using ImageJ. The ratios of the labyrinth to placenta, spongiotrophoblast to placenta, and labyrinth to spongiotrophoblast were used for quantitative analysis.

### Neutral lipid droplet staining

To detect intracellular lipid accumulation, Nile red staining (Bioss, China, D10390) or Oil Red O staining (Beyotime, China, C0157S) was performed on placenta tissues. Slides were washed twice with PBS and then incubated with PBS containing 1 μg/ml of Nile red or Oil Red O staining for 30 min at 37 °C. After incubation, slides were washed three times with PBS. For Oil Red O staining, images were captured under bright field illumination. For Nile red staining, after the initial staining, the slides were further stained with Hoechst (Solarbio) to counterstain the nuclei. Subsequently, the slides were washed with PBS, and images were acquired using an Olympus-microscopy.

### RNA extraction and RT-qPCR

Total RNA was isolated from tissues using TRIzol reagent (Invitrogen, USA, 15596026CN) according to the manufacturer’s instructions. The integrity, quantity, and purity of RNA were examined using NanoDrop 2000 ultraviolet spectroscopy (Thermo Scientific, USA). Then, the mRNA was reversed into cDNAs according to the manufacturer’s protocols (Takara, Japan). Real-time quantitative PCR reactions were then performed in a Bio-Rad CFX Connect Real-Time System (Bio-Rad, USA) using TB Green Premix Ex Taq (Takara). Relative gene expression levels were analyzed using comparative Ct methods where Ct was the cycle threshold number normalized to Actin. The primers are shown in Table S2.

### Cell counting kit-8 assay

Cell viability was assessed using the CCK-8 assay kit (Meilunbio, China, MA0218-3), following the manufacturer's instructions. Briefly, 5,000 cells in 100 μL of culture medium supplemented with 10% FBS were seeded into each well of a 96-well plate and incubated overnight. Subsequently, the cells were treated with a variety of drugs as per the experimental design. After this treatment, the medium was replaced with 100μL of serum-free culture medium and 10 μL of CCK-8 solution was added to each well. The plates were then incubated at 37 °C for 2 h. Samples were analyzed at 450 nm using a microplate reader (Tecan, Untersbergstr, Austria).

### Assessment of lipid peroxidation

Lipid peroxidation was detected as previously described (Shi et al. 2024). Briefly, Cells were stained with 1 µM C11-BODIPY™ 581/591 probe (Thermo Fisher Scientific, USA, D3861) away from light for 30 min at 37 °C, washed 3 times with PBS, and stained with Hoechst (Solarbio). Fluorescence was captured by Olympus-fluorescence microscope. Three random fields per sample from 3 experiments were quantified using ImageJ software.

### Flow cytometric analysis

The cells were incubated with the BODIPY-C11 probe at 37 °C in the dark for 30 min after treatment. Next, cells were washed with PBS and harvested by trypsinization. Then, cells were centrifuged at 1000 rpm for 5 min and resuspended in PBS. Finally, data acquisition was performed on a FACS AriaIII Flowmeter (BD Biosciences), and FlowJo software (version V10; Becton Dickinson, USA) was used for analysis. For quantification of flow cytometry analysis, values of mean fluorescence intensity were determined by imageJ.

### Labile iron detection

FerroOrange fluorescence probe (Dojindo, China, F374) was employed to measure intracellular ferrous iron levels following the manufacturer's protocol. Briefly, 20,000–30,000 cells per well were seeded in a 24-well plate. After treatment with DMSO or 2 μM RSL3 the next day, the culture medium was discarded and washed three times with PBS. Subsequently, 1 μM FerroOrange prepared in PBS was added to each well, and the cells were incubated in the dark for 30 min, followed by Hoechst staining for 5 min. After washing with PBS, the slides were mounted with an anti-fluorescence quencher, and ferrous ion fluorescence was captured using an Olympus fluorescence microscope. Fluorescence quantification was performed using ImageJ software on three random fields per sample from three separate experiments. The AOD was calculated by dividing the IOD by the area of the region. The AOD values were then statistically analyzed to compare the expression levels of labile iron between different groups.

### Transmission electron microscopy

Human placental tissue was fixed with 2.5% glutaraldehyde followed by 1% osmium tetroxide, gradually dehydrated in a gradient of ethylene oxide, embedded in 1:1 acetone: epoxy resin. Finally, ultrathin sections at 50 nm were prepared and double-stained with uranium acetate and lead citrate. The TEM images were acquired at an accelerating voltage of 100 kV, with a resolution of 500 nm and a magnification of 40,000x. The ultrastructure, including the integrity of mitochondrial cristae and outer mitochondrial membrane (OMM), were observed under a transmission electron microscope (Hitachi, Japan).

### Western blotting

Protein extracts were prepared from placental tissues and cells using RIPA lysis buffer supplemented with a 1% phosphatase inhibitor cocktail and 1% protease inhibitor cocktail (Beyotime Biotechnology, China). After centrifugation at 12000 g for 15 min at 4 °C, the protein concentration of the supernatant was normalized and added to Laemmli Sample Buffer (Bio-Rad, USA) and DTT. The prepared lysate was then separated by 10% SDS-PAGE and transferred onto polyvinylidene difluoride membranes (Merck Millipore, Germany). After blocking with 5% nonfat dry milk (Bio-Rad, USA) in Tris-buffered saline containing 0.1% Tween-20 for 1 h at room temperature, the membranes were first probed with primary antibodies (Table S3) overnight at 4 °C and then incubated with the corresponding horseradish peroxidase-conjugated secondary antibody (Proteintech, China) for 1 h at room temperature. Immunoreactive protein bands were developed using enhanced chemiluminescence (ECL) substrate (Advansta, USA, K-12045-D50). The grayscale intensity of each band was determined using ImageJ. The intensity value of the target band was normalized by dividing it by the intensity value of the corresponding loading control band. The results were expressed as relative expression levels.

### Measurement of reduced GSH and malondialdehyde (MDA)

Placental tissues were homogenized in 1 mL of extraction buffer on ice; following centrifugation at 8000 g at 4 °C for 10 min, the supernatant was collected for further analysis. The GSH and MDA content was measured using the GSH assay kit (Beyotime, China, S0053) and the Lipid Peroxidation MDA assay kit (Solarbio, China, BC0025), respectively. In addition, the protein concentration of cellular samples was assayed using a BCA protein assay kit.

### Statistical analyses

Statistical analyses were performed using GraphPad Prism software version 9.0. Before the main analysis, data normality was verified using skewness tests to confirm the assumption of normal distribution. Statistical differences between the two groups were determined using parametric or non-parametric Student’s t-test. For single-variable multiple group comparisons, we used one-way ANOVA followed by Tukey’s multiple comparison test to compare the mean of each column with the mean of every other column. For two-variable multiple group comparisons, we used two-way ANOVA followed by Tukey’s multiple comparison test to compare cell means regardless of rows and columns (Larson 2008; Bewick et al. 2004). All data are presented as the mean ± SEM and a p-value of < 0.05 was considered statistically significant. All experiments were performed at least three times independently.

## Results

### Placenta from GDM patient show elevated GLUT1 expression and signs of ferroptosis

**Fig. 1:**
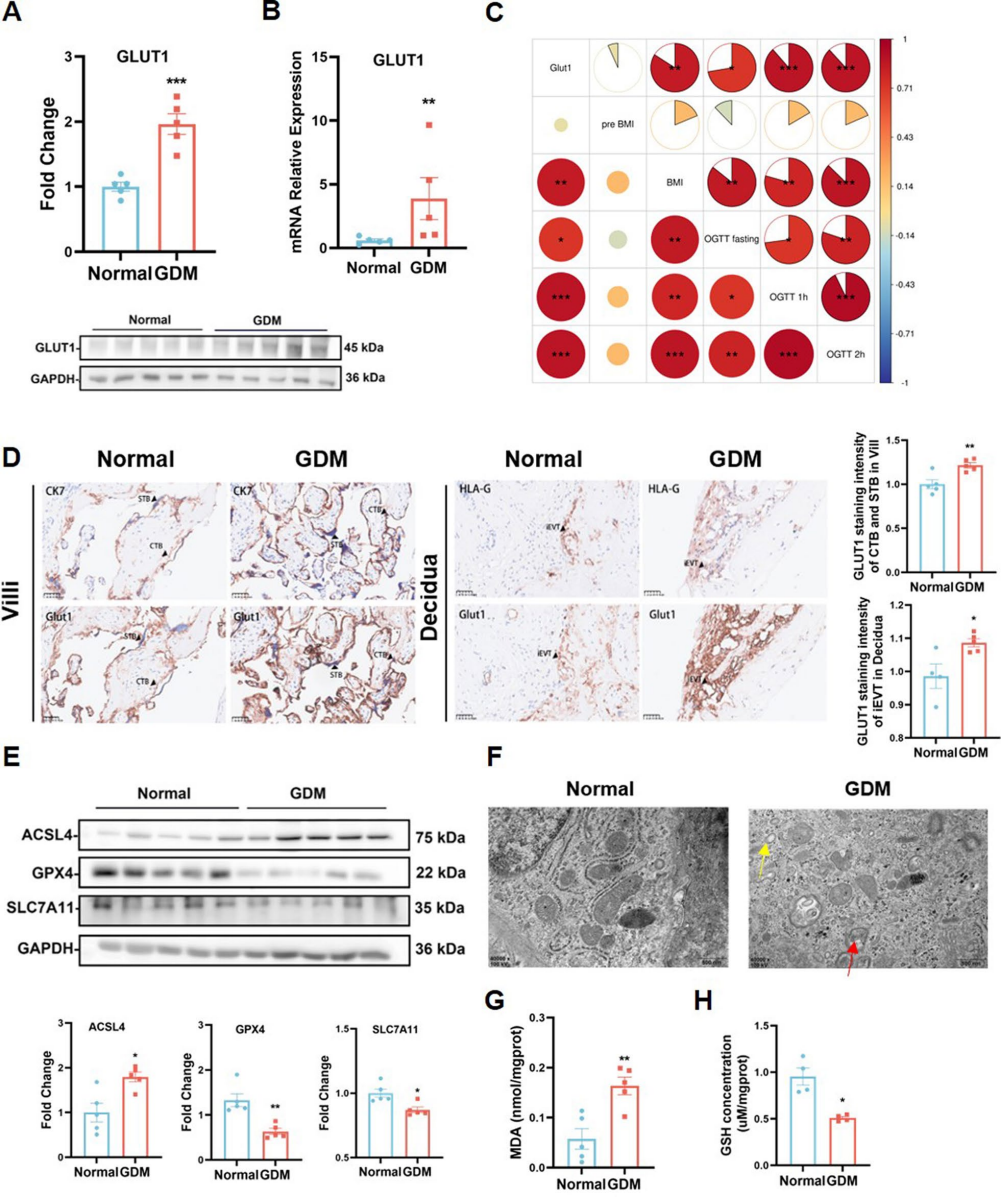
Placenta from GDM Patient show Elevated GLUT1 Expression and Signs of Ferroptosis A total of placental tissues were collected from physiologically normal pregnancies (n = 15) and GDM pregnancies (n = 15). **A** The RT-qPCR analysis of GLUT1 mRNA in normal and GDM term placentas, n = 5, two-tailed t-test. **B** Western blot detection of GLUT1 protein level in normal and GDM term placentas, n = 5, two-tailed t-test. **C** Pearson correlation analysis between placental GLUT1 expression and maternal clinical indicators. **D** IHC staining of GLUT1 in human GDM and normal placentas. Quantification of staining intensity per patient; n = 5. Scale bars: 50 µm, two-tailed t-test. iEVTs and CTBs were identified by HLA-G and CK7 staining, respectively. STB, syncytiotrophoblasts; iEVT, interstitial extravillous trophoblast; CTB, cytotrophoblasts; CK7, cytokeratin 7; HLA-G, human leukocyte antigen G. **E** Representative Western blots of the ferroptosis-associated proteins ACSL4, GPX4, and SLC7A11 in normal and GDM term placentas, n = 5, two-tailed t-test. **F** Representative transmission electron microscopy images showing mitochondrial structure and morphology in normal and GDM term placentas, the red arrows represent ruptured mitochondrial membranes. n = 3, Scale bars: 500 nm. **G** MDA levels were evaluated in human normal and GDM placenta villus, n = 5, two-tailed t-test. **H** GSH concentrations were measured using the corresponding detection kits in normal and GDM term placentas, n = 4, two-tailed t-test. All data are presented as the means ± SEM. *p < 0.05; **p < 0.01; ***p < 0.001

Analysis of GLUT1 expression in placentas from normal and GDM subjects revealed a marked upregulation of both GLUT1 protein and mRNA levels in GDM placentas (Fig. 1A, B). Pearson correlation analysis further underscored a significant positive relationship between placental GLUT1 expression and maternal clinical indicators, including body mass index (BMI) and oral glucose tolerance test (OGTT) results, with deeper red areas in the correlation chart indicating stronger and statistically significant correlations (Fig. 1C). Consistently, immunohistochemistry (IHC) staining confirmed that GLUT1 expression was increased in both syncytiotrophoblasts (STBs) and interstitial extravillous trophoblasts (iEVTs) in GDM placentas when compared to the control group, a finding further verified by immunofluorescence (IF) staining that demonstrated abundant GLUT1 presence in trophoblast cells (Fig. 1D, Supplement 1A). To explore the involvement of ferroptosis in GDM, Western blot analyses revealed a reduction in the expression of key anti-ferroptosis genes GPX4 and SLC7A11, alongside an elevation in ferroptosis-promoting gene ACSL4 in GDM placentas compared to normal counterparts (Fig. 1E). Additionally, RT-qPCR results indicated increased mRNA levels of PTGS2, an independent marker of ferroptosis, as well as TFRC and ACSL4 in GDM placentas (Supplement 1B). Transmission electron microscopy (TEM) revealed hallmark mitochondrial defects associated with ferroptosis, such as the disappearance of mitochondrial cristae and rupture of the outer mitochondrial membrane (OMM) in trophoblast cells of GDM placentas (Fig. 1F). Furthermore, a significant accumulation of malondialdehyde (MDA) was observed, indicating aberrant lipid peroxidation in GDM placentas (Fig. 1G), along with a decline in glutathione (GSH) levels within GDM placenta tissue in comparison to the control group (Fig. 1H). These findings collectively point to a significant involvement of ferroptosis in GDM, alongside the marked upregulation of GLUT1 in placental trophoblast cells, indicating that both processes are critically linked to the pathogenesis of GDM-associated FGR.

### GLUT1 mediates trophoblast sensitivity to ferroptosis under high-glucose conditions

**Fig. 2:**
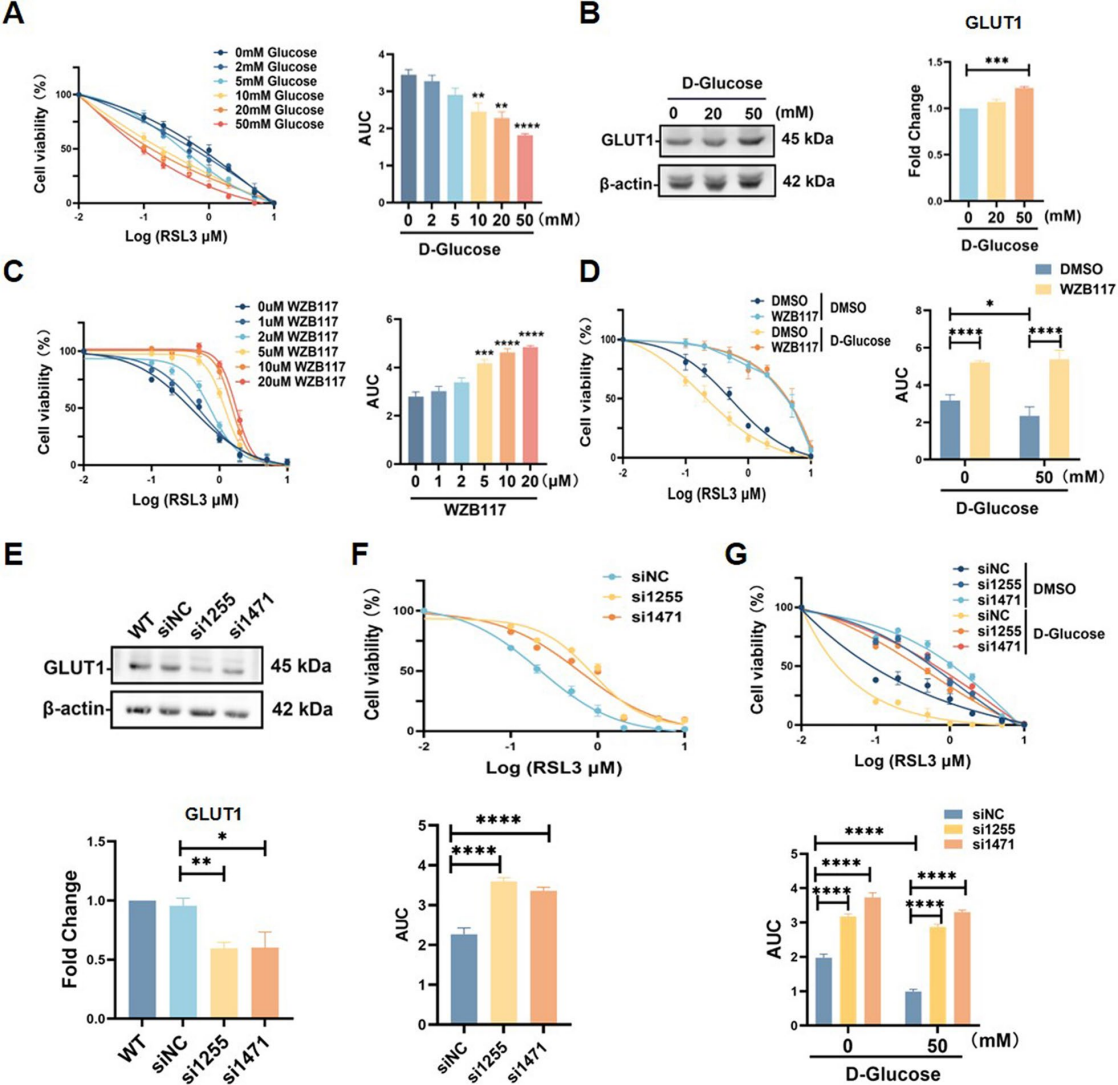
GLUT1 Mediates Trophoblast Sensitivity to Ferroptosis under High-Glucose Conditions **A** HTR8/SVneo cells were treated with indicated concentrations of glucose and RSL3 for 24 h, cell viability was detected by Cell Counting Kit-8 assay, n = 3, Two-way ANOVA and Tukey’s multiple comparison test. **B** Western blot detection of GLUT1 expression under high glucose environment, n = 3, One-way ANOVA, and Tukey’s multiple comparison test. **C** HTR8/SVneo cells were treated with indicated concentrations of RSL3 and WZB117 for 24 h, cell viability was quantitatively assessed using Cell Counting Kit-8 assay, n = 3, Two-way ANOVA, and Tukey’s multiple comparison test. **D** After treating cells with different concentrations of RSL3, cells treated with or without WZB117 (20 μM) under normal or high glucose (50 mM) conditions, cell viability was detected by Cell Counting Kit-8 assay, n = 3, Two-way ANOVA and Tukey’s multiple comparison test. **E** Western blots analysis of GLUT1 in siRNA transfected HTR8/SVneo cells, n = 3, One-way ANOVA, and Tukey’s multiple comparison test. **F** Cells transfected with siRNA were treated with different concentrations of RSL3 for 24 h, then cell viability was determined by Cell Counting Kit-8 assay, n = 3, Two-way ANOVA, and Tukey’s multiple comparison test. **G** HTR8/SVneo cells infected with indicated siRNAs, and then treated with different concentrations of RSL3 under normal or high glucose (50 mM) conditions for 24 h, then cell viability was measured by Cell Counting Kit-8 assay, n = 3, Two-way ANOVA, and Tukey’s multiple comparison test. All data are presented as the means ± SEM. *p < 0.05; **p < 0.01; ***p < 0.001; ****p < 0.0001

Treating cells with GPX4 inhibitor RSL3 is a well-established method for inducing ferroptosis (Bersuker et al. 2019). 25 mM glucose is commonly used to simulate severe hyperglycemia, reflecting the clinical glucose levels (10-25 mM) observed in poorly controlled diabetes, including GDM. For more extreme experimental purposes, 50 mM glucose is sometimes employed to replicate acute hyperglycemic stress or to provoke stronger cellular responses, such as excessive ROS production, mitochondrial dysfunction, and advanced glycation end-product (AGE) formation (3,11). Therefore, we used 50mM as the highest glucose concentration and tested ferroptosis response. Interestingly, when HTR8/SVneo cell was exposed to increasing concentrations of glucose (2 mM, 5 mM, 10 mM, 20 mM, and 50 mM), the sensitivity to RSL3 was enhanced (Fig. 2A). Western blot analysis revealed a substantial increase in GLUT1 protein expression in HTR8/SVneo cells under high glucose conditions (Fig. 2B). This observation led us to further explore GLUT1's role in regulating ferroptosis under both normal and high glucose conditions. Based on the results, we selected the most effective concentration, 50 mM, to establish the high-glucose environment. As shown in Fig. 2C, D, the GLUT1 inhibitor WZB117 significantly reduced RSL3-induced ferroptosis in a dose-dependent manner under both conditions. Additionally, similar results were observed when GLUT1 was knocked down using two different siRNAs (Fig. 2E–G). These findings suggest that GLUT1 promotes ferroptosis in trophoblasts under both normal and high glucose conditions.

### GLUT1 exacerbates lipid peroxidation in trophoblast cells

**Fig. 3:**
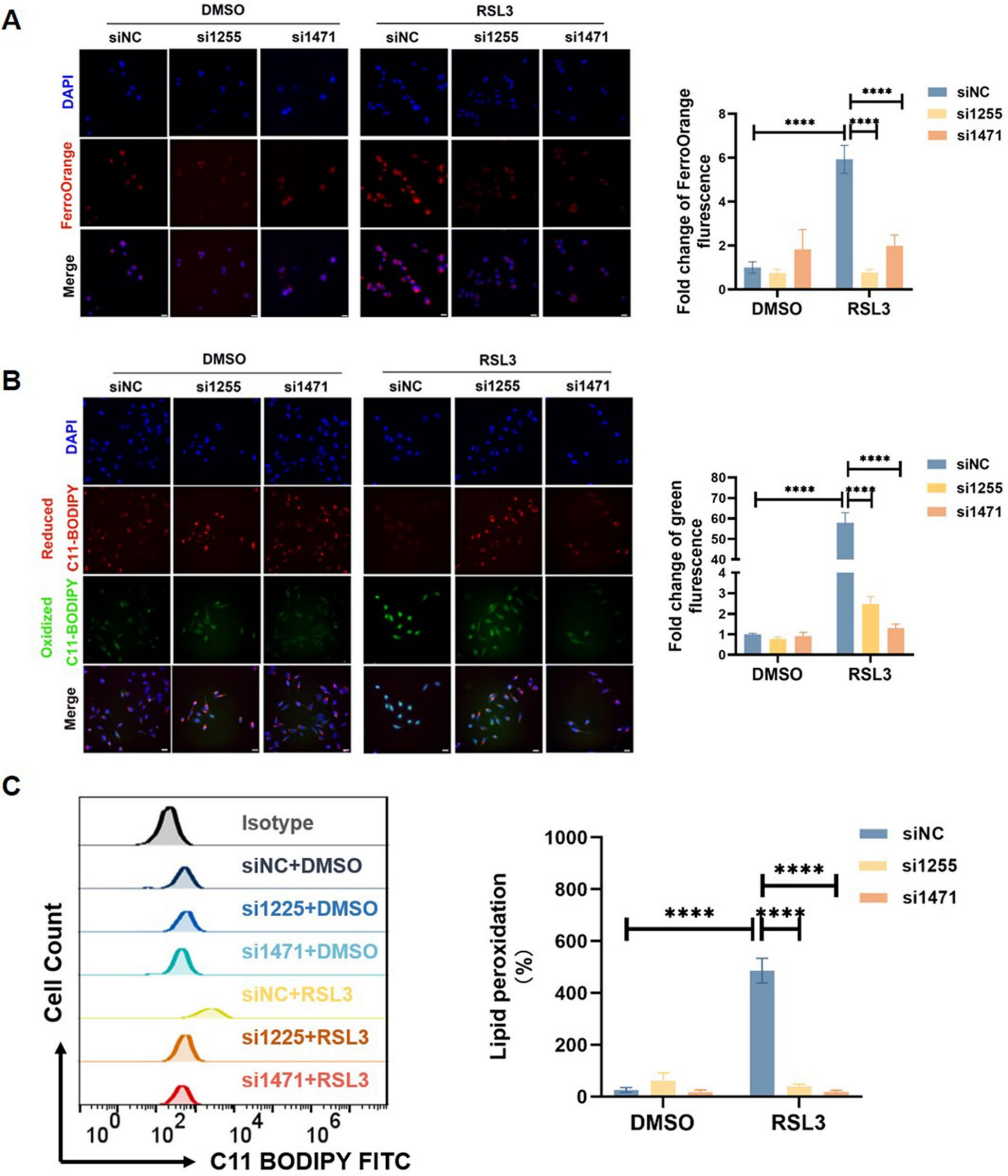
GLUT1 Exacerbates Lipid Peroxidation in Trophoblast Cells **A** Control (siNC) and GLUT1 knockdown (si1255 and si1471) HTR8/SVneo cells were treated with 2 μM RSL3 for 24 h, then intracellular Fe^2+^ was measured by fluorescence microscopy using the FerroOrange fluorescent probe. FerroOrange (red) stains Fe.^2+^ and DAPI (blue) stains the nucleus; n = 3. Scale bar: 20 µm. **B** Control (siNC) and GLUT1 knockdown (si1255 and si1471) HTR8/SVneo cells were treated with 2 μM RSL3 for 24 h, then lipid peroxidation were detected by fluorescence microscopy using the C11 BODIPY 581/591 fluorescent probe. Reduced C11 BODIPY 581/591 (red), oxidized C11 BODIPY 581/591(green), DAPI (blue) stained nucleus, n = 3. Scale bar: 20 µm. One-way ANOVA and Tukey’s multiple comparison test. **C** Control (siNC) and GLUT1 knockdown (si1255 and si1471) HTR8/SVneo cells were treated with 2 μM RSL3 for 24 h, then flow cytometry assay for measuring lipid peroxidation by staining with C11 BODIPY 581/591 fluorescent probe, n = 5. Two-way ANOVA and Tukey’s multiple comparison test. All data are presented as the means ± SEM. ****p < 0.0001

Lipid peroxidation and increased ferrous iron (Fe^2^⁺) levels are key indicators of ferroptosis. To determine whether GLUT1 influences these processes, we used FerrOrange and C11-BODIPY staining methods. As shown in Fig. 3A, B, GLUT1 knockdown led to a significant reduction in both Fe^2^⁺ levels and lipid peroxidation induced by RSL3. This reduction in lipid peroxidation was further confirmed through flow cytometry analysis in GLUT1 knockdown cells treated with RSL3 (Fig. 3C). These findings suggest a mechanistic connection between GLUT1 and ferroptosis, primarily through its effects on lipid peroxidation dynamics.

### GLUT1 regulates lipid metabolism via the AMPK/ACC pathway under high-glucose condition

**Fig. 4:**
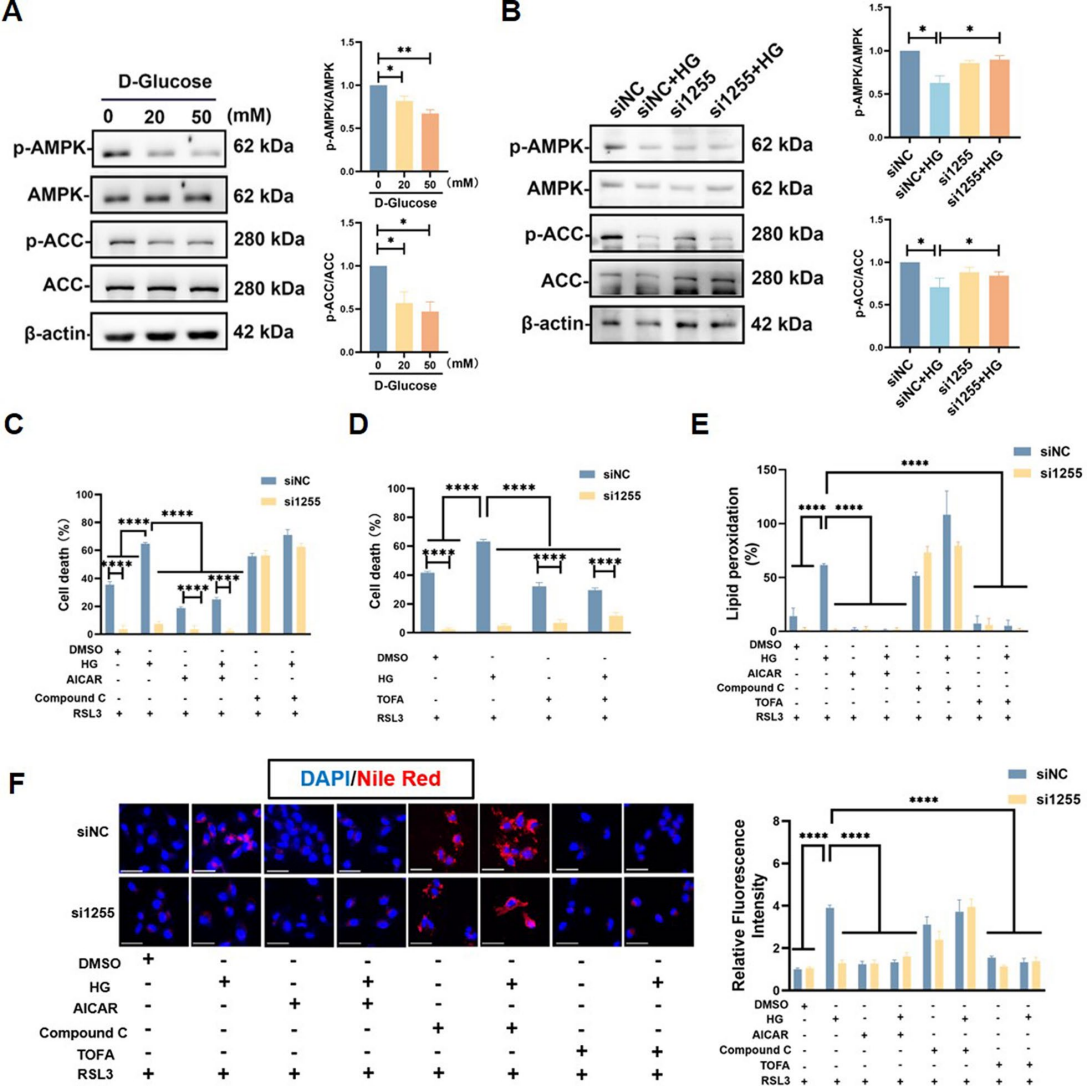
GLUT1 Regulates Lipid Metabolism via the AMPK/ACC Pathway under High-Glucose Condition **A** Western blots detection of p-AMPK, AMPK, p-ACC, and ACC expression under high glucose environment, n = 3, One-way ANOVA, and Tukey’s multiple comparison test. **B** Representative Western blots of the p-AMPK, AMPK, p-ACC, and ACC expression in DMSO and high glucose (50 mM) treated Control (siNC) and GLUT1 knockdown (si1255) HTR8/SVneo cells, n = 3, two-way ANOVA, and Tukey’s multiple comparison test. **C** Cell death measurements in Control (siNC) and GLUT1 knockdown (si1255) HTR8/SVneo cells treated with 50 mM glucose, AICAR (100 μM), Compound C (5 μM) or **D** TOFA (5 μM) cotreated with 2 μM RSL3 for 24 h, **E** flow cytometry assay for measuring lipid peroxidation by staining with C11 BODIPY 581/591 fluorescent probe, **F** Nile red staining of lipid droplet accumulation, n = 3, two-way ANOVA, and Tukey’s multiple comparison test. All data are presented as the means ± SEM. *p < 0.05; **p < 0.01; ****p < 0.0001

To explore how GLUT1 regulates ferroptosis under high-glucose conditions, we examined the activation of the AMPK pathway using Western blot analysis. As shown in Fig. 4A, phosphorylation levels of AMPK and ACC decreased with increasing glucose concentrations. Notably, in high-glucose environment (50 mM), the siNC group exhibited a significant reduction in AMPK and ACC phosphorylation. However, this decrease was not observed following GLUT1 knockdown (Fig. 4B and Supplement 2A). Phosphorylated AMPK is an active form of the enzyme, which phosphorylates ACC, inhibiting its function and consequently reducing the synthesis of polyunsaturated fatty acids (PUFAs). These PUFAs serve as key substrates for lipid peroxidation in ferroptosis. Therefore, under high-glucose conditions, GLUT1-mediated suppression of AMPK and ACC phosphorylation facilitates ferroptosis. Functionally, treatment with AICAR (an AMPK agonist) or TOFA (an ACC inhibitor) increased resistance to RSL3-induced ferroptosis in the high-glucose setting, an effect enhanced by GLUT1 inhibition (Fig. 4C, D). Conversely, this protective effect was reversed by Compound C, an AMPK inhibitor (Supplement 2B and 2C). Flow cytometry analysis of lipid peroxidation using C11 BODIPY 581/591 confirmed increased lipid peroxidation under high-glucose and RSL3 conditions, which was mitigated by GLUT1 knockdown or treatment with AICAR/TOFA. Compound C reversed this protection, restoring lipid peroxidation (Fig. 4E and Supplement 2D). The accumulation of lipid droplets under high-glucose conditions in the control group, and in the knockdown group when treated with Compound C, suggests that the AMPK/ACC pathway is crucial in modulating lipid metabolism during ferroptosis (Fig. 4F and Supplement 2E).

### Inhibition of GLUT1 ameliorates GDM-induced FGR in mice

**Fig. 5:**
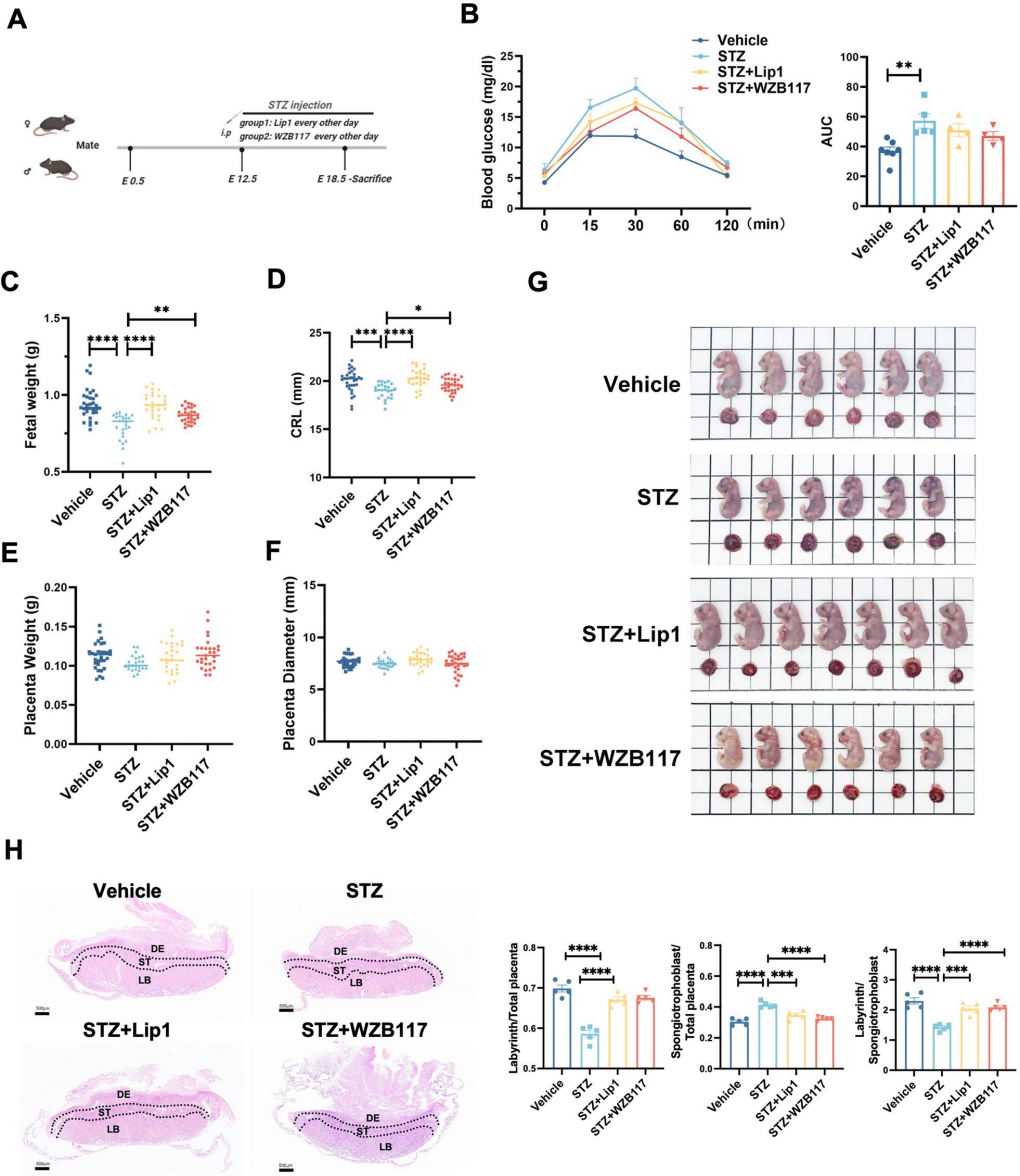
Inhibition of GLUT1 Ameliorates GDM-induced FGR in Mice **A** Schematic illustration of the experimental design. **B** Fasting blood glucose of pregnant mice in Vehicle (n = 7), STZ (n = 5), STZ + Lip1(n = 4), and STZ + WZB117 (n = 4), one-way ANOVA and Tukey’s multiple comparison test. **C** Fetal weight, **D** fetal crown-rump length (CRL), **E** placenta weight, and **F** placenta diameter measured on E18.5 in Vehicle, STZ, STZ + Lip1, and STZ + WZB117 groups, n = 25–35, one-way ANOVA and Tukey’s multiple comparison test. **G** Representative images of the fetuses in Vehicle, STZ, STZ + Lip1, and STZ + WZB117 groups. Scale bars:1 cm. **H** H&E staining of placental sections at E18.5 in in Vehicle, STZ, STZ + Lip1, and STZ + WZB117 groups. labyrinth/placenta ratio, labyrinth/spongiotrophoblast ratio, and spongiotrophoblast/placenta ratio were quantified; n = 5. Scale bars: 500 µm, one-way ANOVA, and Tukey’s multiple comparison test. All data are presented as the means ± SEM. *p < 0.05; **p < 0.01; ***p < 0.001; ****p < 0.0001

Given that GLUT1 expression is increased in GDM patients and promotes ferroptosis in trophoblast cells, we investigated whether inhibiting GLUT1 could alleviate this condition in a GDM induced FGR mouse model, using streptozotocin (STZ). Since E14.5 in mice corresponds to the mid-gestation period in humans, aligning with the clinical timing for GDM screening and diagnosis (Ander et al. 2019). Therefore we chosen to inject STZ at E12.5 of gestation time. Gestational diabetes was induced by administering STZ at embryonic day 12.5 (E12.5), followed by treatment with the ferroptosis inhibitor Lip1 or the GLUT1 inhibitor WZB117 on alternate days from E13.5 to E18.5 (Fig. 5A). At E18.5, blood glucose levels were significantly higher in the STZ group compared to controls, and although Lip1 or WZB117 treatment partially reduced glucose levels, the reductions were not statistically significant (Fig. 5B). STZ exposure also led to significant reductions in fetal weight and crown-rump length, with a slight but non-significant decrease in placental weight and diameter (Fig. 5C–5G). Morphological analysis revealed a marked reduction in the placental labyrinth layer in the STZ group, with altered labyrinth to placenta, spongiotrophoblast to placenta, and labyrinth to spongiotrophoblast ratios (Fig. 5H).

### Inhibition of GLUT1 protects GDM induced FGR in STZ mouse model

**Fig. 6:**
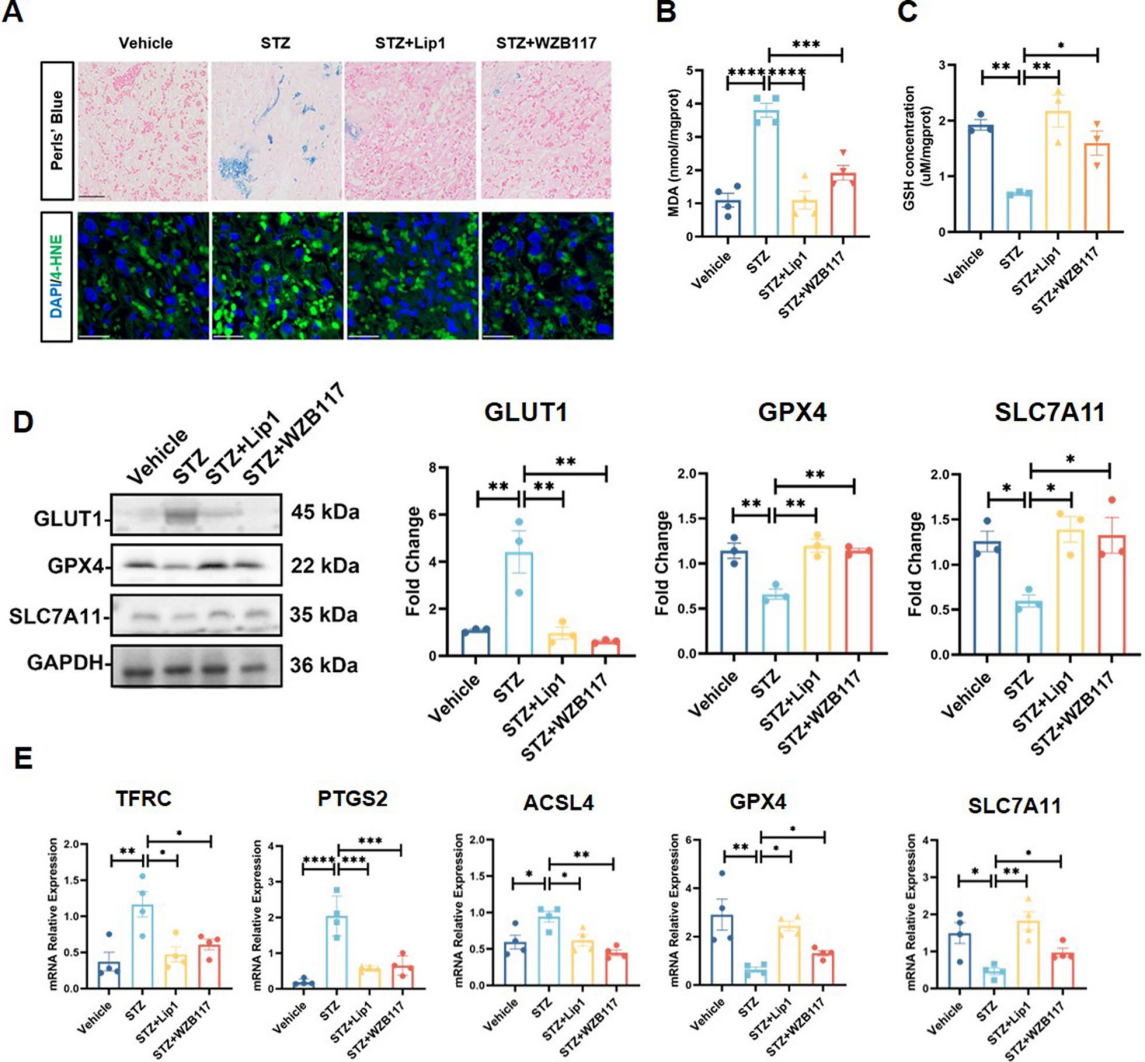
Inhibition of GLUT1 Protects GDM Induced FGR in STZ Mouse Model **A** Perls’ Blue staining and IF staining of 4-HNE of E18.5 placental sections from Vehicle, STZ, STZ + Lip1, and STZ + WZB117 groups; n = 3, Scale bars: 50 µm. **B** MDA concentrations were quantitatively assessed in placental tissues from Vehicle, STZ, STZ + Lip1, and STZ + WZB117 groups, n = 4, one-way ANOVA, and Tukey’s multiple comparison test. **C** GSH concentrations were measured using the corresponding detection kits in placental tissues from Vehicle, STZ, STZ + Lip1, and STZ + WZB117 groups, n = 3, one-way ANOVA, and Tukey’s multiple comparison test. **D** Western blots anaylsis of GLUT1, GPX4 and SLC7A11 expression in placenta tissues from Vehicle, STZ, STZ + Lip1, and STZ + WZB117 groups, n = 3, One-way ANOVA, and Tukey’s multiple comparison test. **E** RT-qPCR determination of mRNA levels of TfRC, PTGS2, GPX4, SLC7A11, and ACSL4 in placentals from Vehicle, STZ, STZ + Lip1, and STZ + WZB117 groups, n = 4, one-way ANOVA and Tukey’s multiple comparison test. All data are presented as the means ± SEM. *p < 0.05; **p < 0.01; ***p < 0.001; ****p < 0.0001

To determine whether the protective effect of the GLUT1 inhibitor WZB117 in the GDM-induced FGR model is linked to its regulation of ferroptosis, we investigated its impact on ferroptosis in vivo, with specific markers detailed in the following sections. First, Perl’s blue staining of the placental labyrinth layer in GDM model mice showed an increase in iron accumulation, which was significantly reduced by treatment with Lip1 and WZB117 (Fig. 6A). Additionally, 4-HNE fluorescence staining and MDA assays indicated heightened lipid peroxidation in the placentas of GDM model mice, a condition that was noticeably improved with Lip1 and WZB117 treatment (Fig. 6A, B). GSH levels were notably lower in the placentas of STZ-treated mice compared to the control group, but this decrease was partially reversed by Lip1 and WZB117 (Fig. 6C). Western blots analysis further revealed that placental GLUT1 expression was upregulated in the STZ model, reinforcing the observation that trophoblast GLUT1 levels increase under high-glucose conditions in vivo. Importantly, Lip1 and WZB117 treatment significantly restored the levels of key proteins involved in ferroptosis, such as GPX4 and SLC7A11, which were diminished in STZ-treated placentas (Fig. 6D). This suggests that inhibiting GLUT1 can protect placentas in GDM from ferroptosis-induced damage. These results were further supported by RT-qPCR analysis, which showed changes in the expression of ferroptosis-related genes, including TfRC, PTGS2, GPX4, ACSL4, and SLC7A11 (Fig. 6E).

### Abnormal AMPK/ACC metabolic pathway in GDM

**Fig. 7:**
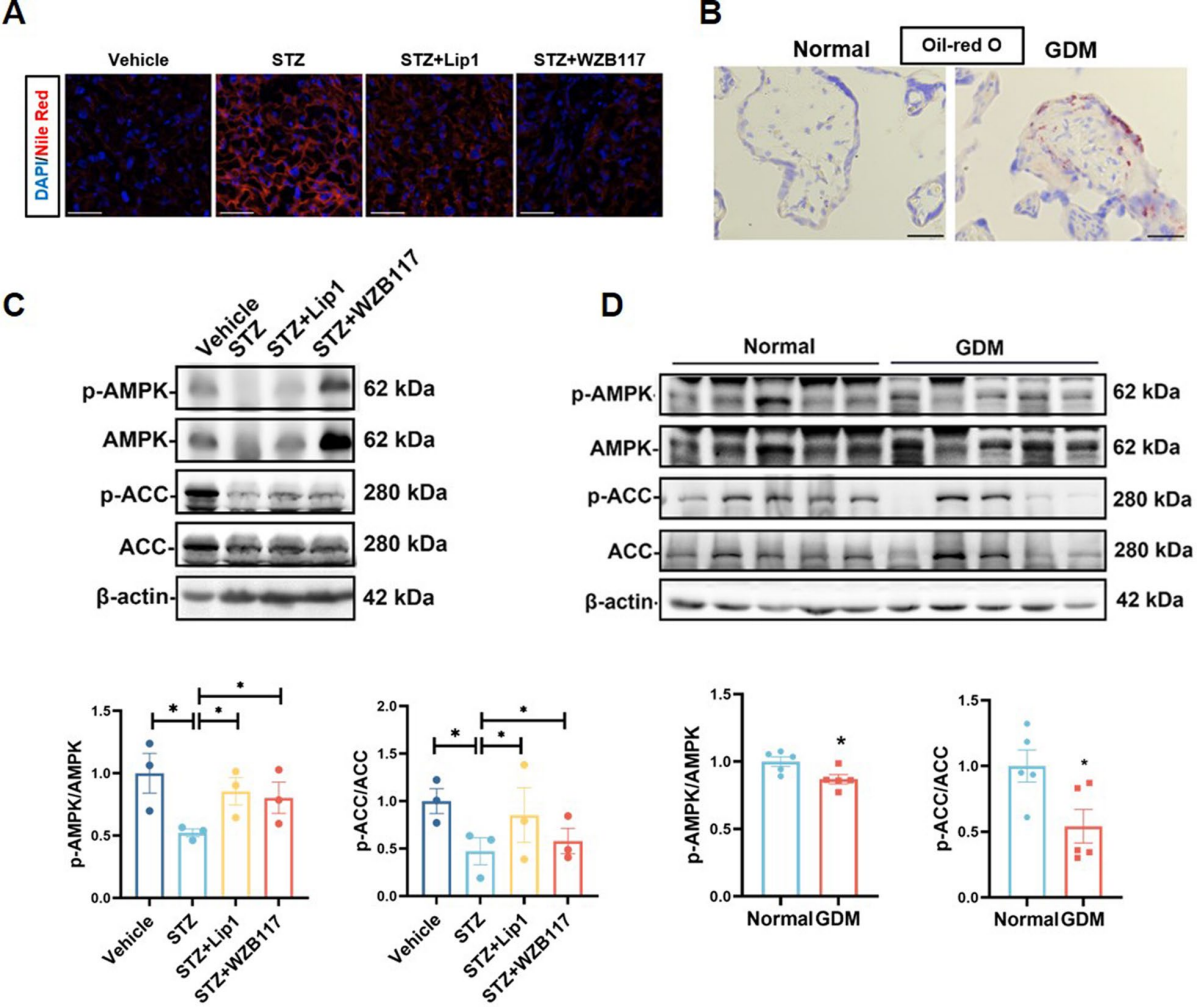
Abnormal AMPK/ACC Metabolic Pathway in GDM **A** The placenta from Vehicle, STZ, STZ + Lip1, and STZ + WZB117 groups were stained with Nile red for fluorescence microscopy, Scale bars: 50 µm. **B** Oil-red O staining of normal and GDM term placentas, n = 3, Scale bars: 50 µm. **C** Representative Western blots of p-AMPK, AMPK, p-ACC, and ACC expression in placenta tissues from Vehicle, STZ, STZ + Lip1, and STZ + WZB117 groups, n = 3, One-way ANOVA, and Tukey’s multiple comparison test. **D** Western blots anaylsis of p-AMPK, AMPK, p-ACC, and ACC expression in normal and GDM term placentas, n = 5, two-tailed t-test. All data are presented as the means ± SEM. *p < 0.05

Nile red staining of mouse placentas revealed a significant increase in lipid droplet accumulation in the STZ-treated group, which was notably reduced with Lip1 and WZB117 treatment (Fig. 7A). Similarly, Oil Red O staining showed an abundance of neutral lipid droplets in the villi of GDM placentas (Fig. 7B). These findings suggest that lipid metabolism is disrupted under GDM conditions. In order to further explore those findings, we examined the expression of the AMPK/ACC pathway in mouse placentas and human placental villi using Western blot analysis. In the STZ model and GDM patients, the phosphorylation levels of AMPK and ACC were lower compared to the control group. However, treatment with Lip1 or WZB117 restored these protein levels (Fig. 7C, D). This indicates that the AMPK/ACC pathway is impaired in GDM, but can be normalized with the inhibition of ferroptosis.

## Discussion

This study sheds light on the crucial role of GLUT1 in driving ferroptosis in trophoblast cells under GDM conditions, with a particular focus on its impact on the AMPK/ACC pathway. Our findings suggest that GLUT1, when upregulated in GDM, interferes with the normal activation of AMPK, leading to a cascade of metabolic disruptions that promote ferroptosis and contribute to placental dysfunction and FGR. Central to this process is the interaction between GLUT1 and AMPK. Normally, AMPK acts as a cellular energy sensor, and upon activation through phosphorylation, it phosphorylates ACC, thereby inhibiting its ability to promote fatty acid synthesis (Pang et al. 2021; Fullerton et al. 2013). This inhibition of ACC is crucial because it prevents the accumulation of fatty acids, which are precursors to lipid peroxides, key drivers of ferroptosis (Guo et al. 2023). However, in GDM, the overexpression of GLUT1 inhibits AMPK phosphorylation, thereby reducing its capacity to phosphorylate and inactivate ACC. As a result, ACC remains active, leading to increased fatty acid synthesis and subsequent accumulation of lipids. These accumulated lipids are highly susceptible to peroxidation, a hallmark of ferroptosis. The enhanced lipid peroxidation observed in trophoblast cells under GDM conditions highlights the pathogenic role of ACC in this process. By promoting fatty acid synthesis, ACC effectively fuels the ferroptotic process, linking metabolic dysregulation directly to cell death in the placenta.

GLUT1 plays a pivotal role in mediating glucose uptake (Jansson et al. 2001), which in turn influences lipid metabolism through the production of acetyl-CoA and subsequent fatty acid synthesis. This connection is particularly relevant in the context of metabolic diseases where GLUT1 is overexpressed, leading to excessive lipid accumulation (Stanirowski et al. 2022). Understanding the mechanisms by which GLUT1-mediated glucose absorption promotes lipid metabolism could inform new therapeutic strategies for metabolic disorders. The role of AMPK in regulating ferroptosis has been well-documented, with studies showing that energy–stress-mediated AMPK activation can inhibit ferroptosis (Lee et al. 2020; Zou et al. 2024), and that the LKB1-AMPK axis negatively regulates ferroptosis by inhibiting fatty acid synthesis (Kottakis and Bardeesy 2012; Li et al. 2020b) Our findings extend these insights by demonstrating that in GDM, GLUT1 upregulation disrupts this protective mechanism, leading to enhanced ferroptosis through ACC activation. These results suggest that targeting GLUT1 may help restore AMPK function and reduce ferroptosis, potentially offering protective effects in GDM pregnancies. In line with our findings of reduced AMPK phosphorylation and increased lipid droplet accumulation in GDM patients, studies also report altered placental phospholipid composition, with elevated levels of arachidonic acid and docosahexaenoic acid (Uhl et al. 2015). These lipids are known to promote ferroptosis (Song et al. 2021), further supporting the role of GLUT1-mediated lipid metabolism dysregulation in GDM-associated FGR.

Notably, our findings highlight that GLUT1 regulates AMPK activity, establishing a crucial link between metabolic regulation and ferroptosis. In addition to the GLUT1-AMPK axis, the mTOR pathway emerges as a potentially significant player in this network (Cork et al. 2018). While direct evidence connecting GLUT1 to mTOR is still lacking, existing studies suggest that GLUT1-mediated glucose uptake might influence metabolic pathways (Hardie et al. 2012), including mTOR activation, which regulates anabolic processes and autophagy (Kim et al. 2011). Importantly, mTOR activation has been shown to suppress AMPK activity, indirectly affecting ferroptosis. Specifically, mTOR hyperactivation can reduce AMPK-mediated phosphorylation of ACC, enhancing the synthesis of polyunsaturated fatty acids (PUFAs), key substrates for lipid peroxidation. Lipid peroxidation, a hallmark of ferroptosis, may be further exacerbated by oxidative stress resulting from impaired autophagy and the accumulation of damaged organelles due to mTOR dysregulation (Myatt and Cui 2004; Gwinn et al. 2008). In the context of GDM-associated fetal growth restriction (FGR), the inhibition of AMPK by GLUT1 and the potential activation of mTOR may together create a metabolic environment conducive to ferroptosis (Mihaylova and Shaw 2011). While the specific role of mTOR in this pathway requires further investigation, our findings underscore the pivotal role of the GLUT1-AMPK axis in regulating ferroptosis. Future studies targeting mTOR activity or AMPK activation may provide novel strategies to mitigate ferroptosis and improve placental health.

The identification of the GLUT1-AMPK axis as a key regulator in ferroptosis offers a potential biomarker pathway for assessing placental dysfunction in GDM patients. Measuring GLUT1 expression levels and AMPK activity in placental tissues may help stratify GDM patients based on their risk of developing fetal growth restriction (FGR). Additionally, oxidative stress markers and lipid peroxidation products associated with ferroptosis, such as MDA and 4-HNE could serve as complementary diagnostic tools. Current standard management of GDM-associated FGR focuses primarily on rigorous glycemic control, dietary interventions, and careful monitoring of fetal growth and placental health (ElSayed et al. 2023). The therapeutic potential of GLUT1 inhibitors would complement, rather than replace, these foundational strategies by specifically addressing oxidative stress at the placental level. Integrating GLUT1 inhibition within the broader management plan for GDM-associated FGR would require a tailored approach, carefully balancing glucose control with the potential benefits of reducing placental oxidative stress and ferroptosis. Future research and clinical trials are necessary to better define the safety, efficacy, and specific applications of GLUT1-targeted therapies, ensuring they fit seamlessly into the multidisciplinary framework essential for managing GDM and optimizing fetal outcomes.

While the potential therapeutic implications of targeting GLUT1 and the AMPK/ACC pathway are promising, it is crucial to approach these findings with caution. The complexity of metabolic regulation in the placenta means that interventions must be carefully designed to avoid unintended consequences. For example, while inhibiting GLUT1 may reduce ferroptosis, it could also impact glucose availability in the placenta, potentially leading to other metabolic complications. Future research should focus on a detailed mapping of GLUT1’s interactions with other metabolic pathways in the placenta, particularly under GDM conditions. The study has several limitations that warrant consideration. First, in the in vivo model, only a GLUT1 inhibitor was employed, leaving the possibility of off-target effects unaddressed. Utilizing a trophoblast-specific knockout model could provide more definitive evidence of GLUT1's role in promoting ferroptosis under GDM conditions. Second, while the STZ model was used to replicate GDM, incorporating a high-fat diet model would serve as a valuable complement, further strengthening the conclusions. Third, although the therapeutic potential of WZB1117 and Lip1 was demonstrated in the GDM induced FGR mouse model, their potential off-target toxicities remain insufficiently evaluated. Fourth, the study could benefit from a larger sample size in both clinical specimens and animal models to bolster the robustness of the findings. Lastly, to complement the siRNA data, employing CRISPR/Cas9 technology for GLUT1 knockout in vitro would provide more definitive support for the results.

In conclusion, this study reveals how GLUT1 upregulation in GDM disrupts the AMPK/ACC pathway, leading to increased fatty acid synthesis and ferroptosis in trophoblast cells. In vivo experiments confirm that inhibiting GLUT1 can restore AMPK function, reduce ferroptosis, and improve fetal outcomes. As our study highlights, the disruption of lipid metabolism and the pathological activation of ferroptosis are pivotal contributors to GDM-associated FGR. Future investigations into the intricate mechanisms governing ferroptosis and its intersection with lipid metabolic pathways hold the promise of unveiling novel therapeutic targets. Such advances could pave the way for innovative strategies to mitigate the adverse outcomes of GDM and improve maternal and fetal health.

## Supplementary Information

Supplementary material 1.

## Data Availability

No datasets were generated or analysed during the current study.

## Abbreviations

GDM: Gestational diabetes mellitus
FGR: Fetal growth restriction
STZ: Streptozotocin
Lip-1: Liproxstatin-1
ROS: Reactive oxygen species
BPA: Bisphenol A
GLUT1: Glucose transporter 1
AMPK: AMP-activated protein kinase
ACC: Acetyl-CoA carboxylase
CRL: Crown-rump length
MDA: Malondialdehyde
PTGS2: Prostaglandin G/H synthase 2
OGTT: Oral glucose tolerance test
STBs: Syncytiotrophoblasts
IEVTs: Interstitial extravillous trophoblasts
TEM: Transmission electron microscopy
OMM: Outer mitochondrial membrane
GSH: Glutathione
