# Supplementary material for: GLUT1 exacerbates trophoblast ferroptosis by modulating AMPK/ACC mediated lipid metabolism and promotes gestational diabetes mellitus associated fetal growth restriction
Source: Mol Med. 2024 Dec 20;30:257. doi: 10.1186/s10020-024-01028-x (PMC11660491; doi:10.1186/s10020-024-01028-x)
Supplement: Supplementary file 1 — Supplementary material 1. [file 10020_2024_1028_MOESM1_ESM.docx]

**Supplementary tables：**

Supplementary Table S1. Patient characteristics used in this study.

| Characteristics | Normal(n=15) | GDM(n=15) | P value |
| --- | --- | --- | --- |
| Maternal age(years) | 29±2.98 | 28.67±2.47 | 0.74 |
| Gestational age(weeks) | 39.18±0.55 | 38.79±0.91 | 0.16 |
| Past or family history of diabetes | No | No |  |
| Height(m^2^)) | 1.56±0.06 | 1.58±0.0.07 | 0.37 |
| Pre-pregnancy weight(kg) | 50.63±7.05 | 58.57±11.00 | 0.02* |
| Gestational weight gain(kg) | 10.27±3.49 | 12.03±2.72 | 0.13 |
| Pre-pregnancy BMI (kg/m^2^) | 20.58±2.69 | 23.14±3.52 | 0.03 * |
| BMI (kg/m^2^) | 24.73±2.92 | 27.90±3.79 | 0.01 * |
| OGTT at second trimester |  |  |  |
| Fasting glucose (mmol/L) | 4.45±0.35 | 4.9±0.62 | 0.02 * |
| 1-hour glucose (mmol/L) | 10.44±1.67 | 7.33±1.30 | ＜0.001**** |
| 2-hour glucose (mmol/L) | 6.03±0.59 | 8.83±1.38 | ＜0.001**** |
| Neonatal weight (g) | 3374±257.44 | 2872.67±141.66 | ＜0.001**** |

GDM: gestational diabetes mellitus; [BMI](https://www.sciencedirect.com/topics/medicine-and-dentistry/body-mass-index), body mass index; OGTT, oral glucose tolerance test. All data are presented with mean ± standard deviation (SD). **P* < 0.05, *****P*＜0.0001.

Supplementary Table S2. Primers used for RT-qPCR

| Primers (human) | Sequences (5' → 3') |
| --- | --- |
| ACSL4-F | GTAATTGGTGGACAGAACATC |
| ACSL4-R | TACTCTCCTGCTTGTAACTTC |
| PTGS2-F | CTGGCGCTCAGCCATACAG |
| PTGS2-R | CGCACTTATACTGGTCAAATCCC |
| TFRC-F | ACCATTGTCATATACCCGGTTCA |
| TFRC-R | CAATAGCCCAAGTAGCCAATCAT |
| GLUT1-F | ATTGGCTCCGGTATCGTCAAC |
| GLUT1-R | GCTCAGATAGGACATCCAGGGTA |
| Actin-F | TGGCACCCAGCACAATGAA |
| Actin-R | CTAAGTCATAGTCCGCCTAGAAGCA |
|  |  |
| Primers(mouse) | Sequences (5' → 3') |
| ACSL4-F | CCTGAGGGGCTTGAAATTCAC |
| ACSL4-R | GTTGGTCTACTTGGAGGAACG |
| PTGS2-F | GCGACATACTCAAGCAGGAGCA |
| PTGS2-R | AGTGGTAACCGCTCAGGTGTTG |
| TFRC-F | GTGGAGTATCACTTCCTGTCGC |
| TFRC-R | CCCCAGAAGATATGTCGGAAAGG |
| GPX4-F | GCAACCAGTTTGGGAGGCAGGAG |
| GPX4-R | CCTCCATGGGACCATAGCGCTTC |
| SLC7A11-F | CTTTGTTGCCCTCTCCTGCTTC |
| SLC7A11-R | CAGAGGAGTGTGCTTGTGGACA |
| Actin-F | TGTGACGTTGACATCCGTAAAG |
| Actin-R | TCAGTAACAGTCCGCCTAGAA |

Supplementary Table S3. Antibodies used for Western blotting

| Antibody name | Brand name | Dilution ratio |
| --- | --- | --- |
| Anti-GPX4 | Abcam | 1:1000 |
| Anti-SLC7A11 | Cell Signaling Technology | 1:1000 |
| Anti-ACSL4 | Abclonal | 1:1000 |
| Anti-GLUT1 | Proteintech | 1:1000 |
| Anti-Actin | Proteintech | 1:1000 |
| Anti-GAPDH  Anti-p-AMPK  Anti-AMPK  Anti-p-ACC  Anti-ACC | Proteintech  Cell Signaling Technology  Cell Signaling Technology  Cell Signaling Technology  Cell Signaling Technology | 1:1000  1:1000  1:1000  1:1000  1:1000 |

**Supplementary figures：**


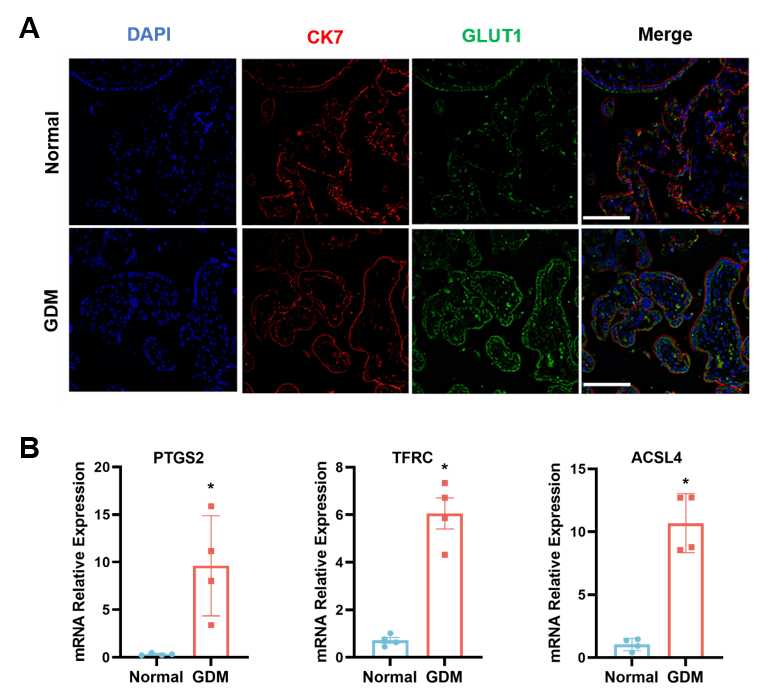


**Figure S1：****Placenta from GDM Patient Show Elevated GLUT1 Expression and Signs of Ferroptosis** (A) IF staining of GLUT1 in human normal and GDM placentas, n=5，Scale bars: 50 µm. (B) mRNA levels of PTGS2, TFRC, and ACSL4 in normal and GDM term placentas determined by qRT-PCR, n=4, two-tailed t-test. All data are presented as the means ± SEM. * *p*< 0.05.


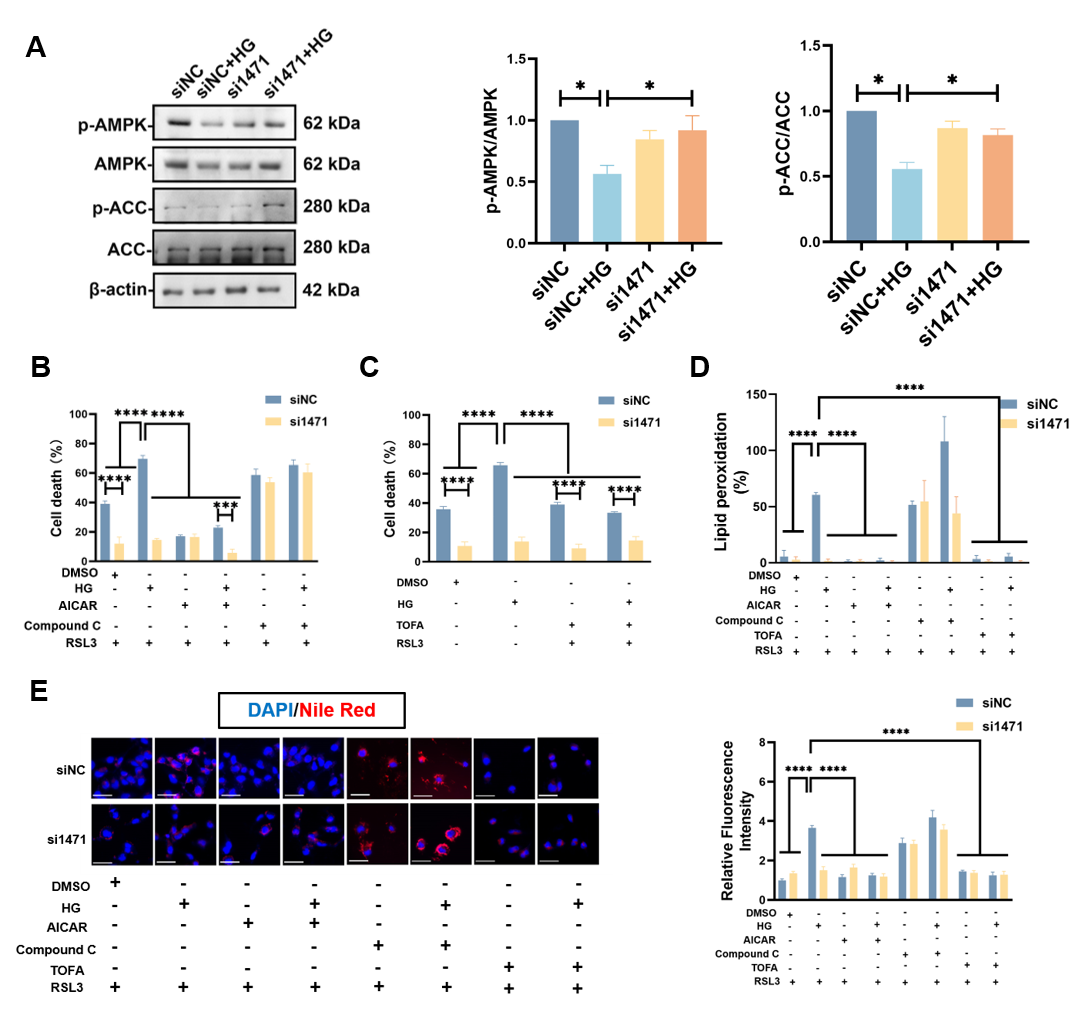


**Figure S2：****GLUT1 Regulates Lipid Metabolism via the AMPK/ACC Pathway under High-Glucose Condition** (A) Representative Western blots of the p-AMPK, AMPK, p-ACC, and ACC expression in DMSO and RSL3 (2μM) treated Control (siNC) and GLUT1 knockdown (si1471) HTR8/SVneo cells, n = 3, two-way ANOVA, and Tukey’s multiple comparison test. (B) Cell death measurements in Control (siNC) and GLUT1 knockdown (si1471) HTR8/SVneo cells treated with 50mM glucose, AICAR (100 μM)，Compound C (5 μM) or (C) TOFA (5 μM) cotreated with 2μM RSL3 for 24h, (D) flow cytometry assay for measuring lipid peroxidation by staining with C11 BODIPY 581/591 fluorescent probe, (E) Nile red staining of lipid droplet accumulation, n = 3, two-way ANOVA, and Tukey’s multiple comparison test. All data are presented as the means ± SEM. * *p* < 0.05; ****p*<0.001; **** *p* < 0.0001.
